# Modern issues of sugar beet (Beta vulgaris L.) hybrid breeding

**DOI:** 10.18699/VJ21.043

**Published:** 2021-07

**Authors:** S.D. Karakotov, I.V. Apasov, A.A. Nalbandyan, E.N. Vasilchenko, T.P. Fedulova

**Affiliations:** Shchelkovo Agrokhim Company, Shchelkovo, Moscow region, Russia; The A.L. Mazlumov All-Russian Research Institute of Sugar Beet and Sugar, vil. VNIISS, Ramonsky district, Voronezh region, Russia; The A.L. Mazlumov All-Russian Research Institute of Sugar Beet and Sugar, vil. VNIISS, Ramonsky district, Voronezh region, Russia; The A.L. Mazlumov All-Russian Research Institute of Sugar Beet and Sugar, vil. VNIISS, Ramonsky district, Voronezh region, Russia; The A.L. Mazlumov All-Russian Research Institute of Sugar Beet and Sugar, vil. VNIISS, Ramonsky district, Voronezh region, Russia

**Keywords:** sugar beet, monogermity, cytoplasmic male sterility, homozygous haploid lines, PCR analysis, сахарная свекла, односемянность, цитоплазматическая мужская стерильность, гомозиготные гаплоидные линии, ПЦР-анализ

## Abstract

High efficiency of the cultivation of unfertilized sugar beet ovules and preparation of haploid regenerants (microclones) of pollinators – maintainers of О-type sterility and MS forms of the RMS 120 hybrid components has been shown. A technological method that accelerates the creation of new uniform starting material is proposed. It speeds up the breeding process two to threefold. The identification of haploid regenerants with sterile cytoplasm in initial populations is of great theoretical and practical importance for breeding, as it facilitates the production of homozygous lines with cytoplasmic male sterility and high-performance hybrids on sterile basis. As shown by molecular analysis, a single-nucleotide polymorphism never reported hitherto is present in the mitochondrial genome of the haploid plant regenerants. It allows identification of microclones as fertile and sterile forms. It has been found that DNA markers of the sugar beet mitochondrial genome belonging to the TR minisatellite family (TR1 and TR3) enable reliable enough identification of haploid microclonal plants as MS- or O-type forms. Fragments of 1000 bp in length have been detected in monogenic forms in the analysis of 11 sugar beet plants cultured in vitro by PCR with the OP-S4 random RAPD primer. Testing of the OP-S4 marker’s being in the same linkage group as the genes responsible for expression of the economically valuable trait monogermity demonstrates its relative reliability. By the proposed method, dihaploid lines (DH) of the male-sterile form and the О-type sterility maintainer of the RMS 120 sugar beet hybrid have been obtained in in vitro culture. These lines are highly uniform in biomorphological traits, as proven under field conditions.

## Abstract (ru)

Показаны высокая эффективность культивирования неоплодотворенных семязачатков растений сахарной свеклы и получение гаплоидных регенерантов (микроклонов) опылителей – закрепителей стерильности О-типа и МС-форм компонентов гибрида сахарной свеклы РМС 120. Предлагается технологический метод, который способствует уменьшению времени создания нового выровненного исходного материала, что ведет к ускорению селекционного процесса. Идентификация гаплоидных регенерантов со стерильной цитоплазмой из исходных популяций имеет важное теоретическое и практическое значение для селекции, так как облегчает задачу создания гомозиготных линий с цитоплазматической мужской стерильностью и высокопродуктивных гибридов на стерильной основе. По результатам проведенного молекулярно-генетического анализа, в митохондриальном геноме гаплоидных растений-регенерантов обнаружен ранее не описанный в литературе однонуклеотидный полиморфизм, позволивший идентифицировать данные микроклоны как фертильные и стерильные формы. Установлено, что ДНК-маркеры митохондриального генома сахарной свеклы, относящиеся к семейству минисателлитов TR (TR1 и TR3), дают возможность с высокой эффективностью выявлять гаплоидные микроклональные растения МС- и О-типа. Установлена информативность маркера OP-S4 для определения раздельноплодных форм. При помощи метода культуры in vitro получены дигаплоидные линии (DH) мужско-стерильной формы и закрепителя стерильности О-типа гибрида сахарной свеклы РМС 120. Линии характеризуются высокой степенью выравненности по биоморфологическим признакам, что было подтверждено в полевых условиях.

## Introduction

Sugar beet (Beta vulgaris L.) is an important source of sucrose, and sugar is one of the essential ingredients in the human diet and a source of readily available energy for the body. The global demand for sugar is increasing at a rate of about 1 Mt (0.5 %) per year, while the population is growing about three times faster. The Russian Federation ranks first in the world in sugar beet planting hectarage (1 million ha), leaving behind such countries as the United States (490 thousand ha), Germany (350 thousand ha), and France (280 thousand ha) (www.fao.org). In recent years, however, approximately 98 percent of areas under sugar beet was planted in Russia with imported seeds of foreign breeding, which has a highly negative impact on the technological and economic sustainability of the whole sugar-beet industry in Russia.

The competitiveness of domestic hybrids depends on the feasibility of unleashing their inherent genetic potential. The use of modern biotechnological and molecular techniques in breeding practice accelerates twofold the development of genetically uniform material, which ensures a high uniformity of root morphology parameters (size, weight, height of head protrusion, depth of the fibrous root system, etc.), as well as sustainable implementation of major commercially valuable traits (monogermity, crop capacity, sugar content, abiotic and biotic stress tolerance during the growing stage, prolonged viability during storage, etc.) during the reproduction. The combination of biotechnology and conventional breeding methods permits one not only to increase the productivity of sugar beet hybrids, but also to improve the quality of seed material. 

One of the major challenges in sugar beet industry is the need to breed monogerm hybrids on the basis of cytoplasmic male sterility (CMS). Spontaneously mutant monogerm plants that served as starting forms for developing monogerm beetroot varieties and components of hybrids were discovered more than 65 years ago (Kolomiyets, 1960; Popov, 1960). The phenotypic polymorphism of multi- and monogerm forms of sugar beet and its genetic control were investigated by many researchers; however, there is no general consensus on the inheritance and manifestation of this trait (Nagamine et al., 1989; Dubrovnaia et al., 2003; Hemayati et al., 2008). 

The monogermity trait can be found in each and every B. vulgaris population. Presumably, choriflowered forms emerge in symflowered populations as a result of natural mutagenesis (Bordonos, 1966; Maletskiy et al., 1991). Earlier, researchers headed by V.F. Savitsky proved the monogenic pattern of inheritance for the М locus, controlling the phenotypic manifestation of multi- and monogermity (Savitsky, 1952). The recessive allele m (monogerm) is responsible for the monogermity trait, and the dominant allele M (multigerm), for multigermity. Monogerm (or choriflowered) genotypes are homozygous for the recessive allele mm. Multigerm (or symflowered) plants are either heterozygous (Mm) or homozygous for the dominant allele (MM ). Deviations from the monogenic inheritance pattern are described in studies conducted by S.I. Maletskiy et al. (1991), who presumed a two-locus model of monogermity inheritance (mmIi). According to S.I. Maletskiy et al. (1991), M is a structural locus, and there is also a regulatory locus, named I (I for inhibitor). S.I. Maletskiy et al. define the dominant alleles of the regulatory locus, which inhibit the development of the symflowered SF (or multigerm) phenotype, as suppressors, or inhibitors, while the recessive alleles of this locus, which do not inhibit the development of the SF phenotype, as enhancers. According to this hypothesis, plants of the choriflowered (monogerm) phenotype should bear recessive alleles of the M locus and dominant alleles of I. More recent papers place the M locus to linkage group 2 on chromosome 4 on the genetic map of B. vulgaris (Schumacher et al., 1997; Amiri et al., 2011). Russian researchers have described a new recessive gene for monogermity, m2. This gene is also mapped on linkage group 2 (Shavrukov, 2000).

Biotechnological methods for the development of double haploids (DH technologies) are currently implemented to obtain homozygous lines and increase the genetic diversity (Dunwell, 2010; Chen et al., 2011; Kikindonov et al., 2016). One of the major challenges in sugar beet breeding is the development of hybrids with the heterosis effect on sterile basis. To obtain these hybrids, monogerm male-sterile (MS) plants are used as a maternal component. Multigerm fertile pollinator plants are the paternal component. To achieve the constancy of the maternal component with regard to monogermity, MS-lines and maintainer lines that maintain the Owen-type (O-type) sterility are usually obtained by prolonged inbreeding during 5–10 generations, which results in inbreeding depression. To overcome this undesirable factor, the in vitro culture of unfertilized ovules is increasingly used in sugar beet breeding nowadays. This technique shortens the time required for producing homozygous genetically stable lines (DH lines, doubled haploids) with regard to such important breeding traits as monogermity, sterility/fertility, etc. The produced dihaploid DH lines require molecular testing of their monogermity, sterility/fertility, etc. at early development stages. 

The objectives of this work are to develop appropriate technologies for producing in vitro sugar beet dihaploid lines and to perform their molecular examination and selection based on monogermity, sterility/fertility, etc.

## Materials and methods

Experiments were conducted with parent components of sugar beet diploid hybrid RMS 120: MS-line and maintainer of О-type sterility RF8 (Ramonskaya fertile). The authors of this hybrid are V.P. Oshevnev, N.P. Gribanova, et al., and the originator is The A.L. Mazlumov All-Russian Research Institute of Sugar Beet and Sugar, hereafter referred to as VNIISS. The RMS 120 hybrid was enlisted to “State Register of Selection Achievements Authorized for Use for Production Purposes” in the Russian Federation in 2008 (https://reestr.gossortrf.ru). 

In our work with tissue culture, unfertilized ovules at the bud development stage were used as explants and 10 % chloramine B solution, as a sterilant. The exposure time was 60 minutes. Ovules isolated in sterile conditions under a microscope were placed into liquid culture medium. The differentiated explants were cultured on solid agar medium (agar 7 g/L) supplemented with auxins and cytokinins in different combinations (6-benzylaminopurine, kinetin, gibberellin, and naphthaleneacetic acid) (Butenko, 1999).

Liquid culture medium supplemented with 1.0 mg/L 6-benzylaminopurine was used for obtaining haploids. They were propagated on agar medium with the addition of gibberellin, 6-benzylaminopurine, and kinetin, 0.2 mg/L each. The haploid material was diploidized by adding 0.01 % colchicine to the culture medium and incubating explants for 36 hours. Rootage developed as microclones were cultured on the medium containing naphthaleneacetic acid (1 mg/L). The regenerants were incubated at 24–26 °С with the daylight time 16 hours, light intensity 5,000 lx, and 70 % relative humidity. The ploidy of samples was determined by flow cytometry (Partec, Germany) according to the recommended protocol (Cousin et al., 2009). 

DNA was extracted from microclones produced via direct regeneration with kits for genomic DNA extraction (Sintol Company, Russia). The quality of DNA samples was tested by electrophoresis in 1 % agarose gel, and concentrations were measured with an HS QubitR Assay Kit (ThermoFisherScientific, USA). The PCR program was: (1) predenaturation at 94 °С for 5 min; (2) 30–33 cycles: denaturation at 94 °С for 30 s, annealing for 40 s, elongation at 72 °С for 60 s; (3) postextension at 72 °С for 3 min. PCR mixture: 1× PCR buffer, 2.5 mM МgCl2, 0.2 mM each dNTP, 1 unit of Taq DNA polymerase, 500 ng of DNA, and 0.5 µmol of primers (see Table).

**Table 1.**
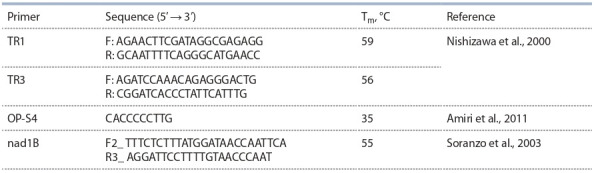
PCR primers

The obtained amplification products were sequenced by the Sanger method on an ABI PRISM 310 Genetic Analyzer (Life Technologies, USA). The PCR amplification products were treated with ColGen kits (Sintol). The results of nucleotide sequence reads were analysed with Mafft software version 7 (Katoh et al., 2016). 

## Results and discussion

**Fig. 1.**
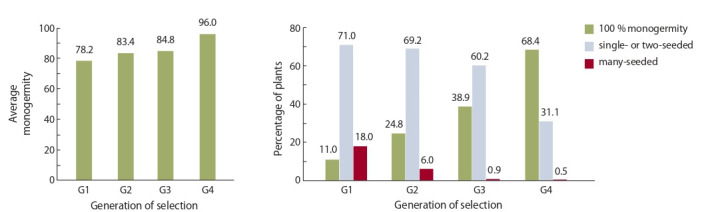
Inheritance of the monogermity trait in the О-type pollinator (Oshevnev et al., 2018).

The study of the monogermity trait during the propagation of fertile pollinators-maintainers of О-type sterility demonstrated an increase in the percentage of descendants with complete (100 %) monogermity from 11 to 68.4 %, with more rigorous selection and rejection of multigerm plants (Oshevnev et al., 2018) (Fig. 1). Also, the mean value of this trait increased from 78.2 to 96 % with increasing the number of selection generations from G1 to G4.

**Fig. 2.**
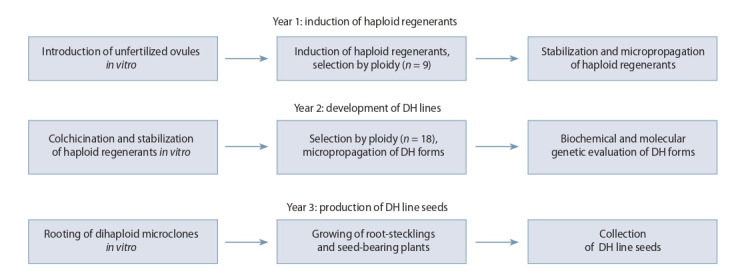
Schematic representation of steps for producing dihaploid sugar beet lines.

The long-term field and laboratory studies conducted at VNIISS allowed developing a technology for producing sugar beet DH lines, which consists of a three-year cycle of biotechnological and breeding steps (Zhuzhzhalova et al., 2020) (Fig. 2). At early stages, regenerants are induced from unfertilized ovules. Plants are selected based on the mono- and multigermity traits and shrub mien (seed-rich multistemmed plants). Sprouts of the central ear of the pleiochasium cluster are generally used as donors of explants. Cytological studies allow selecting genotypes with a high degree of pollen grain fertility and sterility. The selected well-developed haploid regenerants are stabilized by in vitro micropropagation on agar medium. 

The next step includes the diploidization of haploid material by colchicination, stabilization of colchicined regenerants, selection based on biochemical and molecular traits, and formation of in vitro DH lines. 

At the final stages of the technology, dihaploid lines are rooted in vitro, steckling roots and seed-bearing plants are grown in a greenhouse, and seeds of DH lines are collected. The proposed technology produces genetically and morphologically uniform material two to three times faster, omitting the recurrent self-pollination of plants (see Fig. 2).

The selection of genotypes with valuable breeding traits is of great importance in producing homozygous sugar beet lines based on haploids. It is known that sugar beet populations contain plants with normal (N) and sterile (S) cytoplasm. The pollen of N plants is fertile and viable, whereas in S plants it can be either fertile or sterile depending on the interaction between the sterile (S) cytoplasm and recessive alleles (rf1 and rf 2) of the nuclear gene performing the fertility-restoring function. CMS is a result of a complex interaction of certain nuclear and mitochondrial genes (Matsuhira et al., 2012; Chen et al., 2014). This multilocus gene for fertility restoration (Rf  ), a suppressor of mitochondrial genes causing pollen sterility, is one of the best understood genetic factors involved in CMS manifestation (Arakawa et al., 2018).

**Fig. 3.**
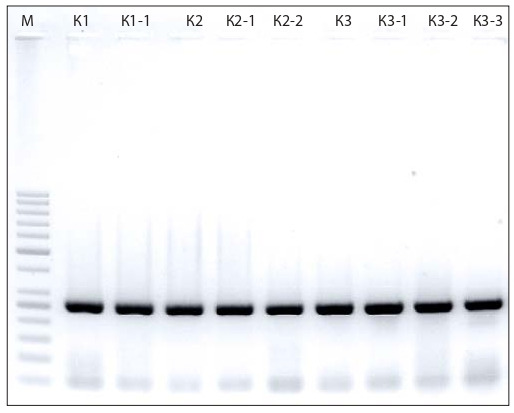
Electrophoretic image of DH regenerant fragments with nad1 gene markers. К1 – control fertile plants; К3 – control sterile plants; К1-1, К2, К2-1, К2-2 – forms with normal (N) cytoplasm; К3-1, К3-2, К3-3 – forms with sterile (S) cytoplasm; М – molecular weight ladder (MassRuler™ DNA marker, 80–1,031 bp, ThermoScientific, USA). Band size: 400 bp

There are also other important genetic factors insufficiently elucidated to date. One of them is the mitochondrial gene nad1 (BevupMp038), which encodes subunit 1 of NADHdehydrogenase in the NADH: ubiquinone oxidoreductase complex. The expression of this gene makes a significant contribution to the interaction between the nuclear and mitochondrial genomes. We employed markers for nad1 to analyze sugar beet dihaploid regenerants, both fertile and sterile (Fig. 3).

**Fig. 4.**
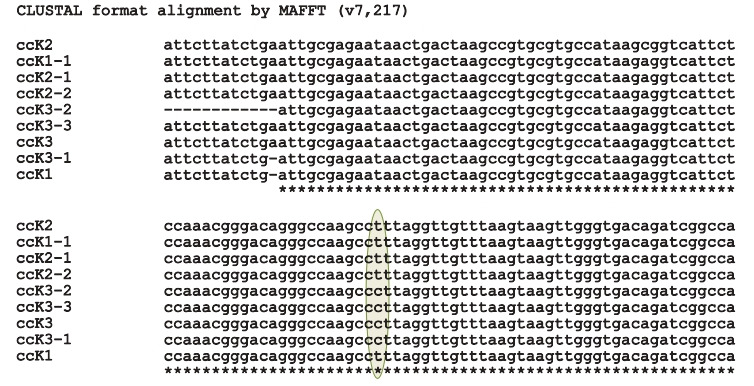
SNP location in the nad1 gene in regenerants. ссK3, ссK3-1, ссK3-2, ссK3-3 – sterile forms; ссK1, ссK1-1, ссK2, ссK2-1, ссK2-2 – fertile forms.

The PCR analysis revealed a 400-bp DNA fragment in all samples. The amplificates were sequenced, and sequence alignment showed their identity except for one single-nucleotide polymorphism. It was shown that in all samples with fertile pollen, i.e. carriers of the nuclear gene dominant allele Rf1, nucleotide C was replaced by T, whereas all haploid sterile forms had only nucleotide C (Fig. 4). 

The detected single-nucleotide substitution is presumed to be significant (non-synonymous), i. e., it can induce an amino acid substitution in the polypeptide, which seems to result in producing a functionally different protein. The presence of this SNP is likely to be related to differences in the CMS trait manifestation in sugar beet.

The genetic polymorphism of the B. vulgaris mitochondrial genome was investigated by using highly variable tandem repeats, or minisatellites. Four tandem repeat loci (TR1, TR2, TR3, and TR4) were found and described in earlier studies of the mitochondrial genomes of sugar beets (Nishizawa et al., 2000; Liu et al., 2017). The TR minisatellite family consists of 30 to 32-bp long sequences arranged in tandems of 2 to 13 in beet genotypes examined (Xia et al., 2020). It was shown that markers TR1 and TR3 are linked to genes controlling CMS (Nishizawa et al., 2000). This finding motivated us to analyze our regenerants with the aforementioned primers. 

**Fig. 5.**
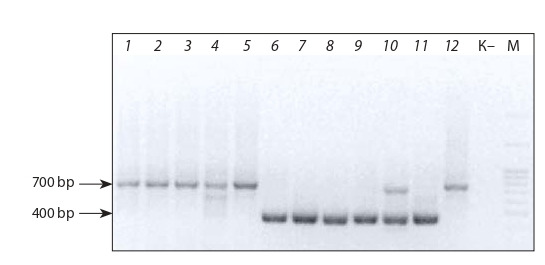
Electrophoretic image of PCR products obtained with the TR1 primer. Lanes: 1–5, 12 – haploid regenerants of О-type pollinator; 6–9, 11 – MS regenerants (haploids); 10 – haploid (mix). М – DNA molecular weight ladder GeneRuler™; 100–3,000 bp (ThermoScientific, USA). “К–” – negative control (sterile water without DNA). Band sizes: 700 and 400 bp

PCR analysis of DNA samples with the TR1 primer revealed 700-bp fragments in O-type haploid forms and 400-bp fragments in MS haploid forms. The sample No. 10 showed both bands (Fig. 5).

As both fragments are amplified in the genome of the sample No. 10, it is rather difficult to say with certainty whether it belongs to the MS- or O-type. Earlier, A.G. Bragin et al. (2012) showed that both N- and Svulg-specific markers can be found in all cytoplasms of plants with both the Owen plasmotype and the plasmotype ensuring the formation of fertile pollen. Their data strongly support the assumption of independent coexistence of mitochondrial genomes of N- and Svulg-types within mitochondria of plants of the same line.

**Fig. 6.**
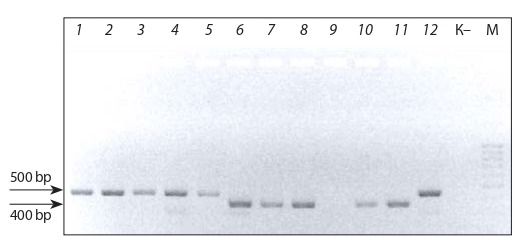
Electrophoretic image of PCR products obtained with the TR3 marker. Lanes: 1–5, 12 – haploid regenerants of О-type pollinator; 6–9, 11 – MS regenerants (haploids); 10 – haploid. М – DNA molecular weight ladder GeneRuler™, 100–3,000 bp (ThermoScientific, USA). “К” – negative control (sterile water without DNA). Band sizes: 500 and 400 bp.

PCR with the primer TR3 revealed 500-bp fragments in O-type haploid forms and 400-bp fragments in MS haploid forms. No DNA fragments were detected in the sample No. 9 (Fig. 6).

Minisatellites are widely used for evaluation of mitochondrial genome polymorphism. This fact may be responsible for the nonuniform patterns of samples No. 9 and 10 obtained by amplification with different TR family minisatellites.

The results of molecular testing suggest that these primers allow early discrimination of haploid regenerants of О- and MS-types, which is of theoretical and practical significance for breeding. Exceptions are the samples No. 10 (two fragments when amplified with TR1) and No. 9 (no amplification product with TR3).

As mentioned above, the crucial breeding trait of sugar beet is monogermity, regulated by the recessive allele of the M-m gene. As with mitochondrial genes, the genes (loci) that control the monogermity trait have not been mapped precisely (Shavrukov, 2000). However, a locus linked to this trait in F1 and F2 populations has been identified. Scientists outside Russia tested 297 single-stranded decamer RAPD primers with an F2 population of monogerm and multigerm sugar beet hybrids. The nearest genetic marker (closer than 50 cM) linked to the monogermity gene was OP-S4 (Amiri et al., 2011).

**Fig. 7.**
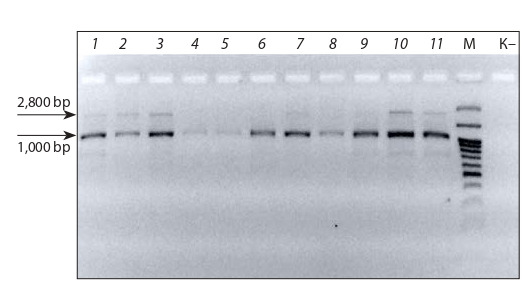
Electrophoretic resolution of PCR products obtained with the OP-S4 primer. Lanes: 1–3 – О-type; 4, 5 – HP (heterosis pollinator); 6–11 – MS. М – DNA molecular weight ladder GeneRuler™, 100–3,000 bp (ThermoScientific, USA). “К–” – negative control (sterile water without DNA). Band sizes: 1,000 and 2,800 bp.

In our PCR experiments, 11 samples of sugar beet obtained in vitro with the OP-S4 primer produced 1000-bp fragments. A second fragment of about 2,800 bp was seen in some samples (No. 1, 2, 3, 10, and 11). DNA fragments from genotypes No. 4 and 5, which are multigerm according to the data from breeders (V.P. Oshevnev, N.P. Gribanova), were barely seen (Fig. 7). 

The results obtained in testing primer OP-S4, belonging to the same linkage group as the gene for monogermity, do not presume reliable ranking of samples according to the monogermity trait at the stage of haploid microclones. This may be related to the low specificity and high sensitivity to conditions of the reaction, characteristic of RAPD primers. The identification of homozygous monogerm genotypes requires a more comprehensive analysis with a larger number of sugar beet plant samples and molecular markers with higher specificity. 

## Conclusion

In this study, an approach to the accelerated development of doubled lines (homozygotes) as components of highly productive hybrids was designed. Seeds were obtained from four B. vulgaris DH lines and used for the propagation of highest quality seeds of the male-sterile form of the RMS 120 hybrid component. Molecular analysis detected an SNP in the genomes of the haploid regenerants, and this SNP allowed the discrimination of fertile and sterile forms. It is shown that mitochondrial minisatellite markers TR1 and TR3 enable classification of haploid regenerants as MS- or O-type forms. Analysis of 11 monogerm and multigerm plants obtained in vitro by PCR with the RAPD primer OP-S4 revealed 1000-bp long fragments in monogerm regenerants. 

By combining biotechnological and molecular methods with traditional breeding techniques, new breeding material can be produced to develop indigenous new-generation sugar beet hybrids.

## Conflict of interest

The authors declare no conflict of interest.
